# Zebrafish In-Vivo Screening for Compounds Amplifying Hematopoietic Stem and Progenitor Cells: - Preclinical Validation in Human CD34+ Stem and Progenitor Cells

**DOI:** 10.1038/s41598-017-12360-0

**Published:** 2017-09-21

**Authors:** Guruchandar Arulmozhivarman, Martin Kräter, Manja Wobus, Jens Friedrichs, Elham Pishali Bejestani, Katrin Müller, Katrin Lambert, Dimitra Alexopoulou, Andreas Dahl, Martin Stöter, Marc Bickle, Nona Shayegi, Jochen Hampe, Friedrich Stölzel, Michael Brand, Malte von Bonin, Martin Bornhäuser

**Affiliations:** 1 Department of Hematology/Oncology, Medical Clinic and Policlinic I, University Hospital, Dresden, Germany; 2 Institute of Biofunctional Polymer Materials, Leibniz Institute for Polymer Research, Max Bergmann Center of Biomaterials, Dresden, Germany; 3 German Cancer Research Center (DKFZ), Heidelberg, Germany; 4 German Consortium for Translational Cancer Research (DKTK), partner site, Dresden, Germany; 5 Deep Sequencing Group SFB655, Biotechnology Center, Technical University of Dresden, Dresden, Germany; 6 Max-Planck Institute of Molecular Cell Biology and Genetics, Dresden, Germany; 7 Department of Hematology, University Hospital Essen, University of Duisburg, Essen, Germany; 8 DFG-Center for Regenerative Therapies Dresden (CRTD) – Cluster of Excellence, Technical University of Dresden, Dresden, Germany

## Abstract

The identification of small molecules that either increase the number and/or enhance the activity of human hematopoietic stem and progenitor cells (hHSPCs) during *ex vivo* expansion remains challenging. We used an unbiased *in vivo* chemical screen in a transgenic (*c-myb:EGFP*) zebrafish embryo model and identified histone deacetylase inhibitors (HDACIs), particularly valproic acid (VPA), as significant enhancers of the number of phenotypic HSPCs, both *in vivo* and during *ex vivo* expansion. The long-term functionality of these expanded hHSPCs was verified in a xenotransplantation model with NSG mice. Interestingly, VPA increased CD34^+^ cell adhesion to primary mesenchymal stromal cells and reduced their *in vitro* chemokine-mediated migration capacity. In line with this, VPA-treated human CD34^+^ cells showed reduced homing and early engraftment in a xenograft transplant model, but retained their long-term engraftment potential *in vivo*, and maintained their differentiation ability both *in vitro* and *in vivo*. In summary, our data demonstrate that certain HDACIs lead to a net expansion of hHSPCs with retained long-term engraftment potential and could be further explored as candidate compounds to amplify *ex-vivo* engineered peripheral blood stem cells.

## Introduction

Mature blood cell lineages originate from a pool of self-renewing hematopoietic stem cells (HSCs) and are an attractive source for stem-cell-based therapies like hematopoietic stem cell transplantation (HSCT) that offer a potential cure for various malignant (leukemia, lymphoma, and myeloma) and non-malignant (aplastic anemia) hematologic disorders. Currently, bone marrow (BM), umbilical cord blood (UCB), and peripheral blood from G-CSF (granulocyte-colony stimulating factor) treated donors are the major sources of stem cells for transplantation, and peripheral blood stem cell transplantation (PBSCT) is the most common and widely used procedure in the clinical setting^1^. However, issues regarding the yield of transplantable HSCs still prevail, especially in the context of UCB transplantation^2^, despite the recent increase in the number of suitable donors and the success of haploidentical HSCT^3^. As low HSC numbers at transplantation have been associated with greater incidence of graft failure, delayed hematopoietic recovery, slow immune reconstitution, and early mortality, even in PBSCT recipients^4^, protocols that facilitate the *ex vivo* expansion of HSCs represent an important step to overcome these limitations. Further, efficient *ex vivo* expansion of genetically modified HSCs, obtained using novel gene editing techniques, can potentially be applied in patients with inborn genetic diseases (e.g. hemoglobinopathies)^5^.

Numerous attempts have been made to identify conditions and/or chemicals that allow the *ex vivo* expansion of functional hematopoietic stem and progenitor cells (HSPC), including cytokine cocktails, feeder layer of mesenchymal stromal cells (MSCs), and proteins or chemicals (e.g. notch ligand, aryl hydrocarbon receptor antagonists, PGE_2_, all-trans retinoic acid, and other epigenetic modulators)^6–13^. However, amplification of HSPCs is not necessarily associated with preservation of HSPC function as some studies have reported loss of self-renewal capabilities, measured by the long-term repopulating capacity of these cells^14^. While certain procedures for *ex vivo* expansion have been shown to retain HSC function and clinical trials have attested to the feasibility of this approach^15^, successful hematopoietic recovery after HSC transplantation not only relies on self-renewal and differentiation capacity but also on homing to the bone marrow and subsequent lodging in hematopoietic stem cell niches^16^. Such migration and lodging of HSCs in specific niches are tightly regulated processes that are controlled by the expression and function of various molecules, including integrins (VLA-4, VLA-5, and LFA-1), selectins (P- and E-selectin), and certain chemokines (SDF-1)^17^.

In this study transgenic *c-myb:EGFP* zebrafish were used to screen and identify small molecules that modulated HSPC activity^18^. Histone deacetylase inhibitors (HDACIs), namely, valproic acid (VPA), resminostat, and entinostat, significantly increased HSPC numbers, and their functional relevance was validated by analyzing runx1^+^ expression in the zebrafish embryos. HDACIs also produced similar effects in human HSPCs as human CD34^+^ cells could be extensively expanded *in vitro* using various HDACIs, especially, VPA. *In vivo*, even though VPA-expanded CD34^+^ HSPCs displayed impaired homing to the bone marrow of immunodeficient mice that resulted in reduced short-term engraftment as monitored by peripheral blood donor chimerism, they however, retained their long-term engraftment potential and maintained their differentiation ability both *in vitro* and *in vivo*. These results imply that HDACIs, particularly VPA, can be used for the *in vitro* expansion of G-CSF mobilized hHSPCs, but their use in clinical transplantation protocols should consider impaired homing and lower short-term-engraftment.

## Results

### HDACIs increase c-myb^+^ HSPC number and *runx1* expression in zebrafish embryos

**Figure 1:**
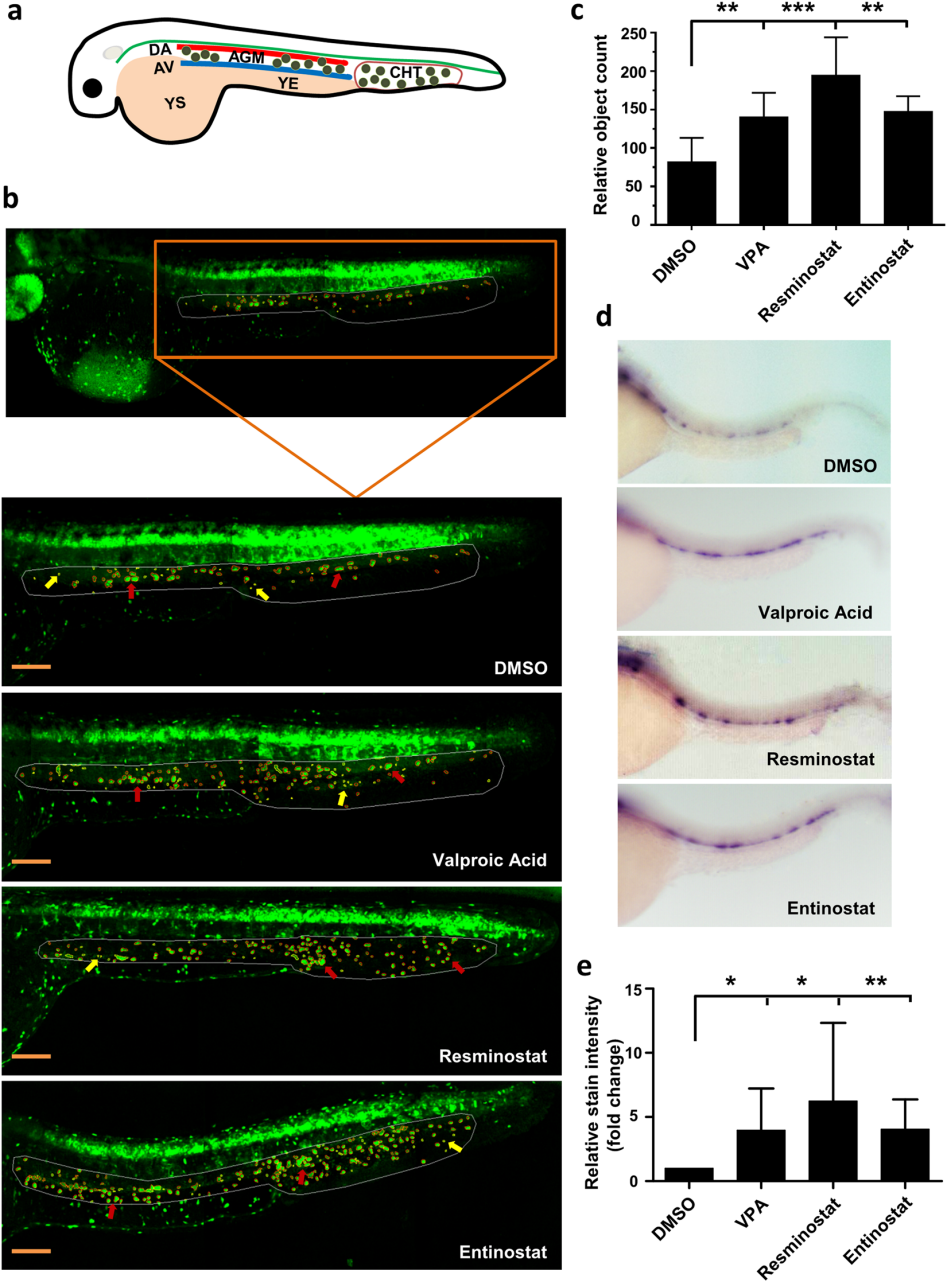
HDACIs increase c-myb^+^ HSPC number and *runx1* expression in zebrafish embryos. (**a**) Schematic representation of HSPC development in the AGM and CHT regions of a zebrafish embryo. YS – yolk sac; YE – yolk extension; DA – dorsal aorta; AV – axial vein; AGM – aorta-gonad-mesonephros; CHT – caudal hematopoietic tissue. Small green circles between the DA and AV and in CHT regions represent HSPCs. (**b**) Image based identification of c-myb^+^ cells in the AGM and CHT region identified HDACIs (valproic acid, resminostat, and entinostat) as enhancers of HSPC cell-count at 36 hpf (Bars = 200 µm). The region-of-interest (AGM and CHT) marked by white line and the c-myb^+^ cells are marked by red circle (indicated by red arrows) and the false positive objects that are excluded from the quantification are marked by yellow circle (indicated by yellow arrows). (**c**) Quantification of relative number of c-myb^+^ cells in the AGM and CHT region showing increased cell-count after 40 µM VPA, resminostat or entinostat treatment compared to DMSO (n = 5). (**d**) Validation of identified hits through whole-mount *in situ* hybridization for *runx1* expression. (**e**) Quantification of *runx1* relative stain intensity shows significantly higher *runx1* expression in HDACI treated fish compared to DMSO controls. Intensity was calculated using area under curve analyses in ImageJ. Single images were split into 5 regions of interest (ROIs) and intensity was normalized to background signal. SD displays deviation among 5 ROIs in one image (n = 3). Data are shown as mean ± SD, *p < 0.05; **p < 0.01; *** p < 0.001.

A recently developed semi-automated imaging assay^18^ was used on transgenic zebrafish embryos expressing *c-myb:EGFP* in HSPCs to screen 550 compounds and identify small molecules that modulate HSPC activity. In zebrafish hematopoiesis, long-term HSCs occur in the aorta-gonad-mesonephros (AGM) at approximately 30 hours post fertilization (hpf) and migrate to the caudal hematopoietic tissue (CHT) region, colonize the thymus, and finally translocate to the kidney marrow, which is the equivalent of mammalian bone marrow^19^ (Fig. 1a). *c-myb* is expressed in the cells of the AGM and CHT regions in zebrafish during hematopoiesis^20,21^. In the assay, embryos were exposed to compounds at concentrations of 20 or 40 µM and between 12 and 36 hpf. Compared to DMSO-treated controls, three HDACIs, namely valproic acid (VPA), resminostat, and entinostat, significantly increased the number of c-myb^+^ cells in the AGM and CHT regions (DMSO 93 ± 4, VPA 137 ± 22, resminostat 194 ± 29, entinostat 150 ± 19, p < 0.001 for all; Fig. 1b and c). These observations were validated by whole-mount *in situ* hybridization (WISH) for *runx1* on wild type embryos as *runx1*, a transcription factor, is an essential regulator of definitive hematopoiesis and is consistently expressed in HSCs at all sites of embryonic and adult hematopoiesis^22^. All three HDACIs triggered an increase in runx1^+^ cells in the AGM compared to minimal expression in control embryos (Fig. 1d,e), suggesting that HDAC inhibition by VPA, resminostat, or entinostat leads to the *in vivo* expansion of HSPCs in zebrafish.

### HDAC inhibitors increase zebrafish hematopoietic cell engraftment efficiency

**Figure 2:**
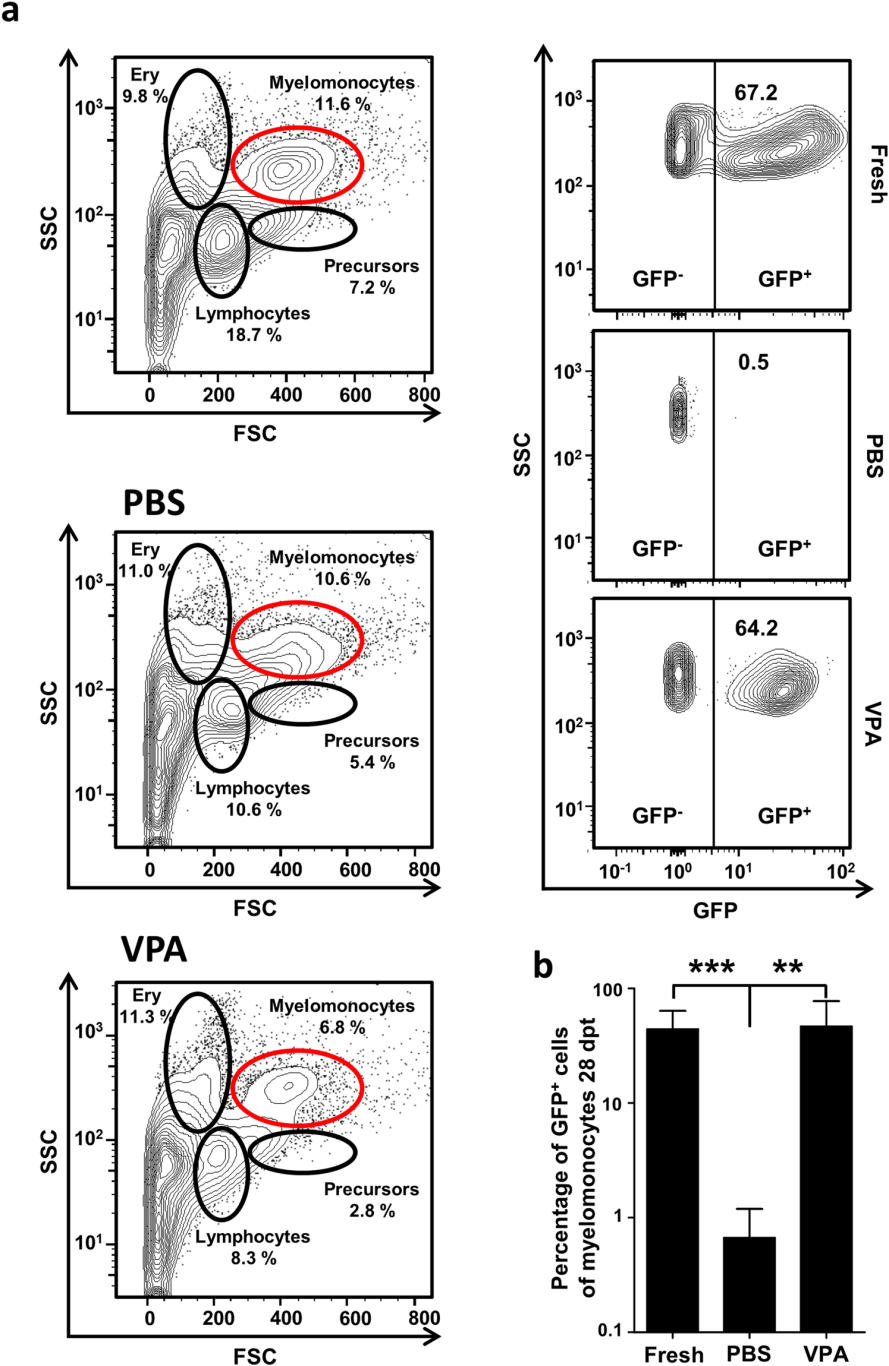
*Ex vivo* VPA treatment preserves engraftment capacity of whole kidney marrow cells. (**a**) A representative plot showing gating strategy used to determine the degree of chimerism in the recipient and the FACS plot of PBS and VPA treated conditions. (**b**) PBS-treated control donor WKM cells failed to engraft the kidney of the recipient, but VPA treated cells demonstrated an engraftment capacity similar to that of freshly isolated cells (uncultured; n = 5 per group). Data are shown as mean ± SD, **p < 0.01, ***p < 0.001.

Of the identified hits, VPA was chosen for validation using an *ex vivo* transplantation procedure. Zebrafish adult whole kidney marrow (WKM) cells from *Tg(ubi:GFP)* animals^23^ were treated with VPA (50 µM) or PBS (control) *in vitro*, transplanted into irradiated Casper^24^ adult zebrafish (Fig. S1). This incubation time was chosen based on data from pilot experiments that showed a sharp reduction in cell viability after longer time-periods of *ex vivo* culture. After 28 days, the WKM cells from recipient Casper fish were analyzed by flow cytometry for engraftment of donor cells (Fig. 2a). VPA treatment significantly increased WKM cell engraftment compared to PBS treatment (donor chimerism 46.5 ± 26.8% vs. 0.7 ± 0.2; p = 0.0056; Fig. 2b), suggesting that *ex vivo* VPA treatment maintains the stem cell potential of WKM cells and increases the engraftment capability after transplantation in this model.

### HDACIs increase CD34^+^ and CD34^+^CD90^+^ cell populations in human G-CSF mobilized peripheral blood stem cells

**Figure 3:**
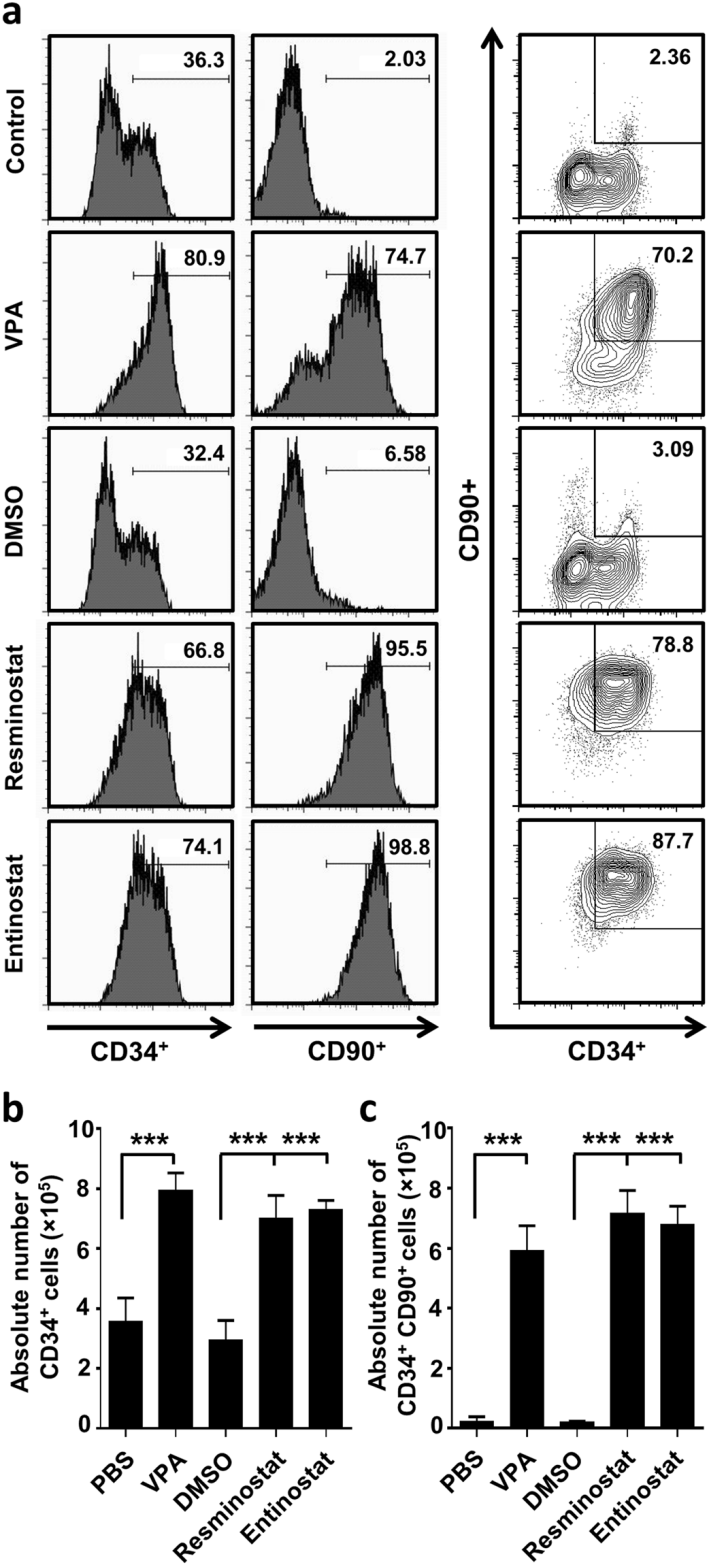
*Ex vivo* expansion of G-CSF mobilized CD34^+^ HSPCs treated for 5 days with HDACIs. (**a**) Representative flow-cytometry analysis of CD34 and CD90 expression after 5 d of *ex vivo* HDACI treatment. (**b**) Absolute cell numbers of CD34^+^ cells. (**c**) Absolute cell numbers of CD34^+^CD90^+^ cells. Both CD34^+^ and CD34^+^CD90^+^ cells were significantly increased after 5 days treatment with VPA (1 mM), resminostat (1.5 µM) or entinostat (1.5 µM) compared to controls (PBS and DMSO; n = 5). Data are shown as mean ± SD, ***p < 0.001.

Next, the effects of HDACIs on human CD34^+^ G-CSF-mobilized peripheral blood stem cells (purity >95%) were analyzed by treating them *in vitro* with VPA, resminostat, or entinostat for five consecutive days in the presence of cytokines. The number and phenotype of the cultured cells were assessed at the end of the treatment period. The proportion of CD34^+^ cells was 79.4 ± 2.4%, 70.2 ± 2.8%, and 72.2 ± 2.2% for VPA, resminostat, and entinostat, respectively, compared to 35.6 ± 3.2% and 29.4 ± 3.3% for PBS- or DMSO-treated controls (p < 0.001 for all; Fig. 3a). Importantly, the fraction of CD34^+^CD90^+^ cells, a population known to be enriched in repopulating cells^25^, was higher in VPA, resminostat, or entinostat treated samples (59.3 ± 3.4%, 71.5 ± 3.8%, and 67.9 ± 4.3%, respectively) compared to PBS- or DMSO-treated cells (2.2 ± 0.7% and 2.0 ± 0.2%, respectively (p < 0.001 for all; Fig. 3a). All three HDACIs also amplified the starting cell numbers compared to controls and resulted in significantly higher absolute cell numbers of both CD34^+^ and CD34^+^CD90^+^ cell populations after 5 days of *in vitro* treatment (Fig. 3b and c). Thus, HDACIs identified from the zebrafish screen expanded phenotypic human HSPCs *in vitro*. As VPA exerted similar effects as the other two compounds but had lower toxicity over a broader range of concentrations, all subsequent experiments on human HSPCs were performed with VPA alone.

### VPA increases attachment of hHSPCs on MSCs during *ex vivo* expansion

**Figure 4:**
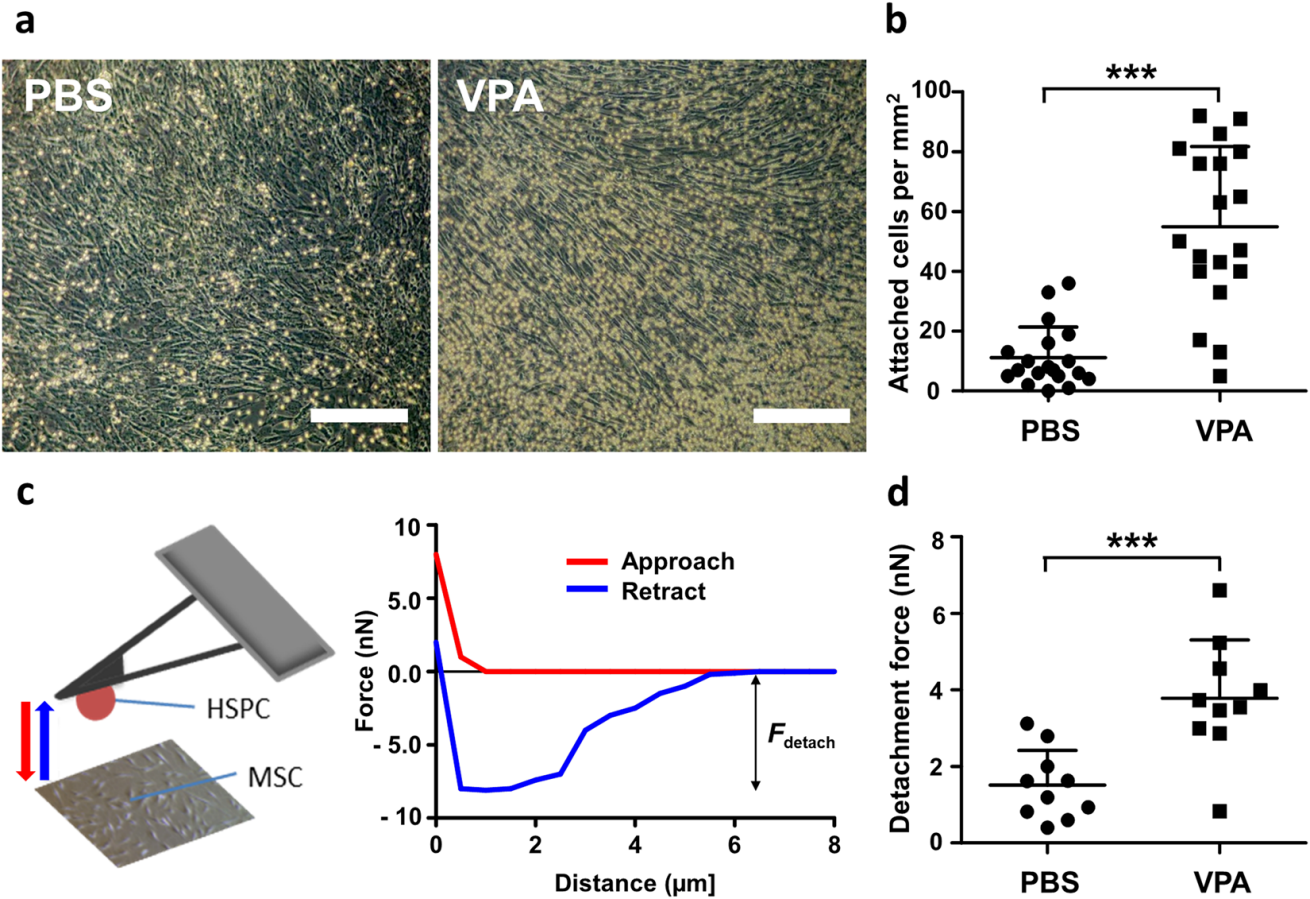
*Ex vivo* VPA-expanded CD34^+^ cells exhibit increased adhesion to MSCs. (**a**) Freshly isolated CD34^+^ cells were seeded onto a confluent MSC layer. Representative images after 5 days of co-culture with MSCs showed an increase in the number of VPA-treated adherent cells (right) compared to control cells (left). Images were taken after sufficient washing with PBS. (Scale bar: 250 µm). (**b**) In the adhesion assay, a significantly higher number of VPA expanded cells were attached to MSCs compared to control (n = 4). (**c**) Schematic representation of atomic force microscopy-based single-cell force spectroscopy used to measure adhesive strength of VPA treated and control cells. **(d)** Plot showing detachment force measurement after 5 days of VPA or PBS (control) treatment. Adhesive strength of VPA treated cells was 2–3 fold higher than control (n = 3). Data are mean ± SD, ***p < 0.001.

As MSCs are one of the supporting factors of HSPC maintenance^26^ and proliferation^11^, we investigated if VPA influences the interaction between hHSPCs and MSCs *in vitro*. Similar to suspension cultures, VPA induced hHSPC expansion in co-cultures with MSCs. In addition, a higher density of hHSPCs were attached to the MSC layer in the VPA-treated group than the control group (Fig. 4a), and 3-fold greater number of hHSPCs were attached to MSCs in the VPA-treated group compared to controls (Fig. 4b). These findings prompted us to investigate the adhesion strength between VPA-treated CD34^+^ cells and MSCs using atomic force microscopy-based single-cell force spectroscopy (AFM-SCFS) under defined conditions of contact force and time. The adhesion between hHSPCs and the substrate-bound MSC was quantified during retraction (Fig. 4c). In accordance with the bulk assays, higher detachment forces, i.e. stronger cell-cell interactions, between HSPCs and MSCs were measured in VPA-treated cells compared to PBS-treated controls (Fig. 4d). These observations suggest that VPA-treated CD34^+^ cells are more adhesive and that this adhesiveness can result in greater interaction with MSCs. Thus, VPA-induced expansion of phenotypic HSPCs *in vitro* is also associated with enhanced attachment to MSCs.

### VPA treatment preserves self-renewal capacity and differentiation potential

To determine whether VPA-expanded cells retained their differentiation capacity, we performed colony forming unit (CFU) assays and compared their clonogenicity to freshly isolated human CD34^+^ cells from GCS-F mobilized blood. VPA-treated cells formed significantly fewer total number of colonies compared to the PBS treated cells (Fig. S3a). This difference was mainly due to an increase in BFU-E (burst forming unit-erythroid) colonies in the PBS-treated CD34^+^ cells as there were no statistically significant differences in the total number of CFU-G, CFU-M, CFU-GM, and CFU-GEMM populations between the VPA- and PBS-treated CD34^+^ cells. These observations imply that VPA treatment does not affect clonogenicity and differentiation capacity of the hHSPCs (Fig. S3b). We also analyzed the cobblestone area forming cell (CFAC) potential of *ex vivo* expanded CD34^+^ cells to characterize their *in vitro* stem cell functions and found no significant differences between VPA-treated and control cells (Fig. S3c).

### VPA suppresses CXCR4 expression and HSPC migration toward SDF-1 *in vitro*

**Figure 5:**
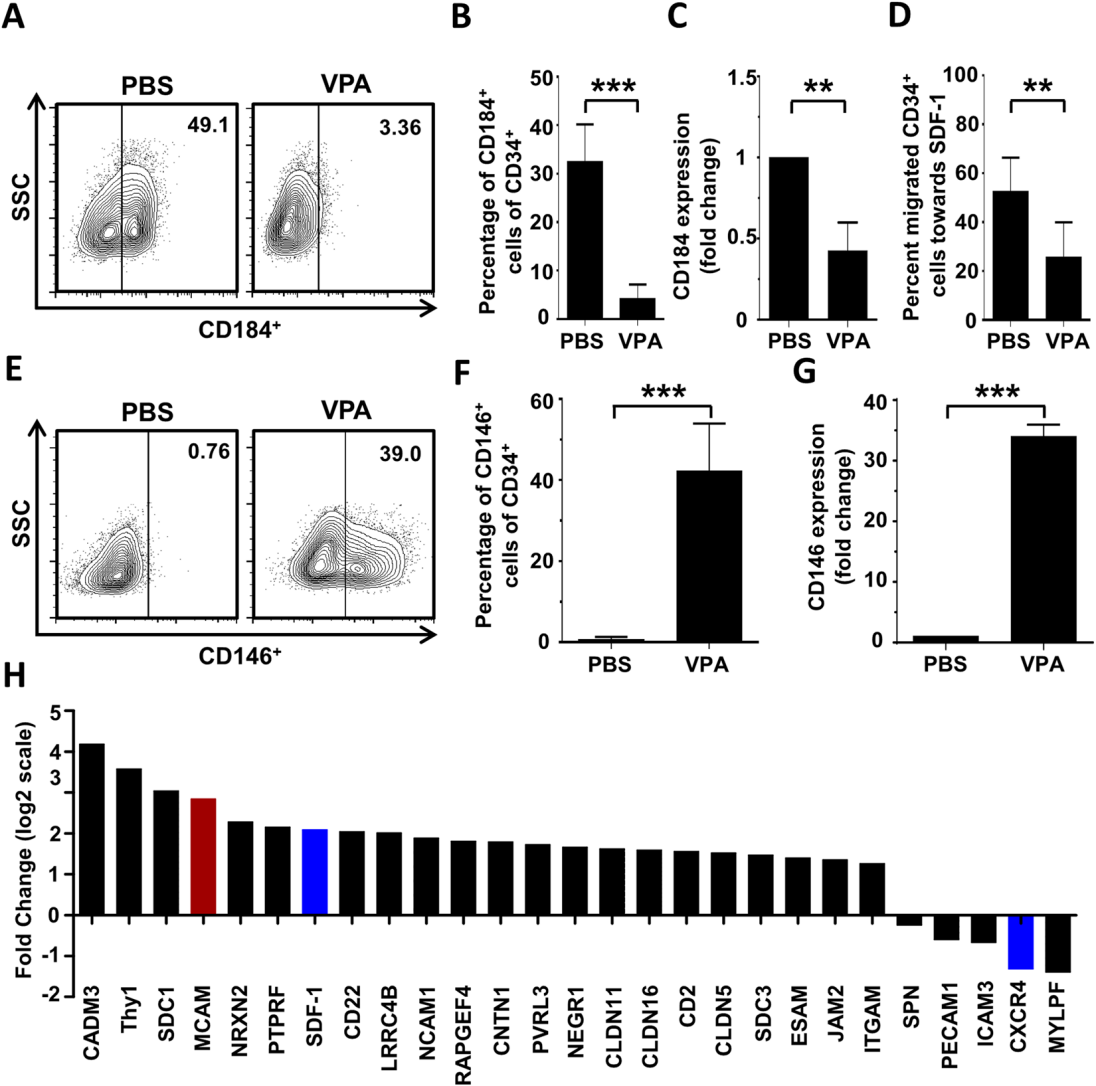
Valproic acid affects adhesion of HSPCs and suppresses their migration toward SDF-1 *in vitro*. G-CSF mobilized CD34^+^ HSPCs were treated *in vitro* for 5 days with VPA or PBS and analyzed for the expression of molecules that are involved in cell adhesion and migration. The functional consequence of VPA-treatment on the migratory capacity toward SDF-1 was also evaluated. (**a**) Representative dot-plot of CD184 (CXCR4) expression on the cell surface of CD34^+^ cells as determined by flow cytometry. (**b**) VPA-treatment significantly reduced the expression of CXCR4 on the cell surface of CD34^+^ cells compared to control cells (n = 3), measured by flow cytometry. (**c**) Reduced CXCR4 expression was confirmed by quantitative PCR (n = 3). (**d**) Trans-well migration assay showed that VPA-treatment significantly reduced the migration capacity of CD34^+^ cells toward an SDF-1 gradient (100 ng/ml) compared to control cells (n = 4). (**e**) Representative plot of CD146 (MCAM) expression on the cell surface of CD34^+^ cells as determined by flow cytometry. (**f**) Flow cytometric analysis showed that VPA-treatment significantly increased surface expression of MCAM on CD34^+^ cells compared to controls (n = 4). (**g**) Significantly higher expression of MCAM in VPA expanded CD34^+^ cells was verified by quantitative PCR (n = 4). (**h**) RNA sequencing revealed that VPA-treatment substantially changed the expression of molecules involved in cell adhesion and migration in CD34^+^ cells compared to control cells, including CXCR4 and MCAM (n = 4). Data are mean ± SD, **p < 0.01, ***p < 0.001.

Successful HSC transplantation depends on effective HSPC homing and engraftment in the bone morrow after transplantation, and stromal-derived factor-1 (SDF-1, CXCL12) and its receptor CXCR4 (CD184) play essential roles^27^. To investigate migration and homing potential of *ex vivo* VPA-expanded hHSPCs, we measured CXCR4 expression using flow cytometry (Fig. 5a) and found a drastic reduction in CXCR4 expression upon VPA treatment compared to control cells (Fig. 5b). This reduction was confirmed by real-time PCR (Fig. 5c) and verified by RNA-seq data (Fig. 5h). RNA-seq data also revealed that VPA treatment induced SDF-1 expression in CD34^+^ cells (Fig. 5h). The functional relevance of these findings was tested by quantifying the migration capacity of VPA-expanded CD34^+^ cells toward SDF-1 using a transwell migration assay where we detected significantly lower numbers of migratory CD34^+^ cells compared to PBS-treated control hHSPCs (Fig. 5d).

Next, we used flow cytometry In order to identify other molecular pathways that could mediate the increased adhesiveness of VPA-treated cells and found that the adhesion molecule CD146 (MCAM) was expressed on 40% of VPA-treated CD34^+^ cells while control cells did not express this molecule (Fig. 5e and f). Quantitative PCR analysis (Fig. 5g) and RNA-seq (Fig. 5h) also showed significantly elevated MCAM transcript levels in VPA-treated cells compared to PBS-treated controls. However, RNA-seq data also showed that VPA induced the expression of several cell adhesion molecules along with MCAM (Fig. 5h). Thus, VPA not only decreases CXCR4 expression on CD34^+^ cells that is associated with reduced migratory potential toward SDF-1, but also triggers the upregulation of specific adhesion molecules including MCAM, which probably mediate the VPA-induced increase in the adhesion capacity.

### VPA treatment reduces bone marrow homing efficiency of CD34^+^ cells

**Figure 6:**
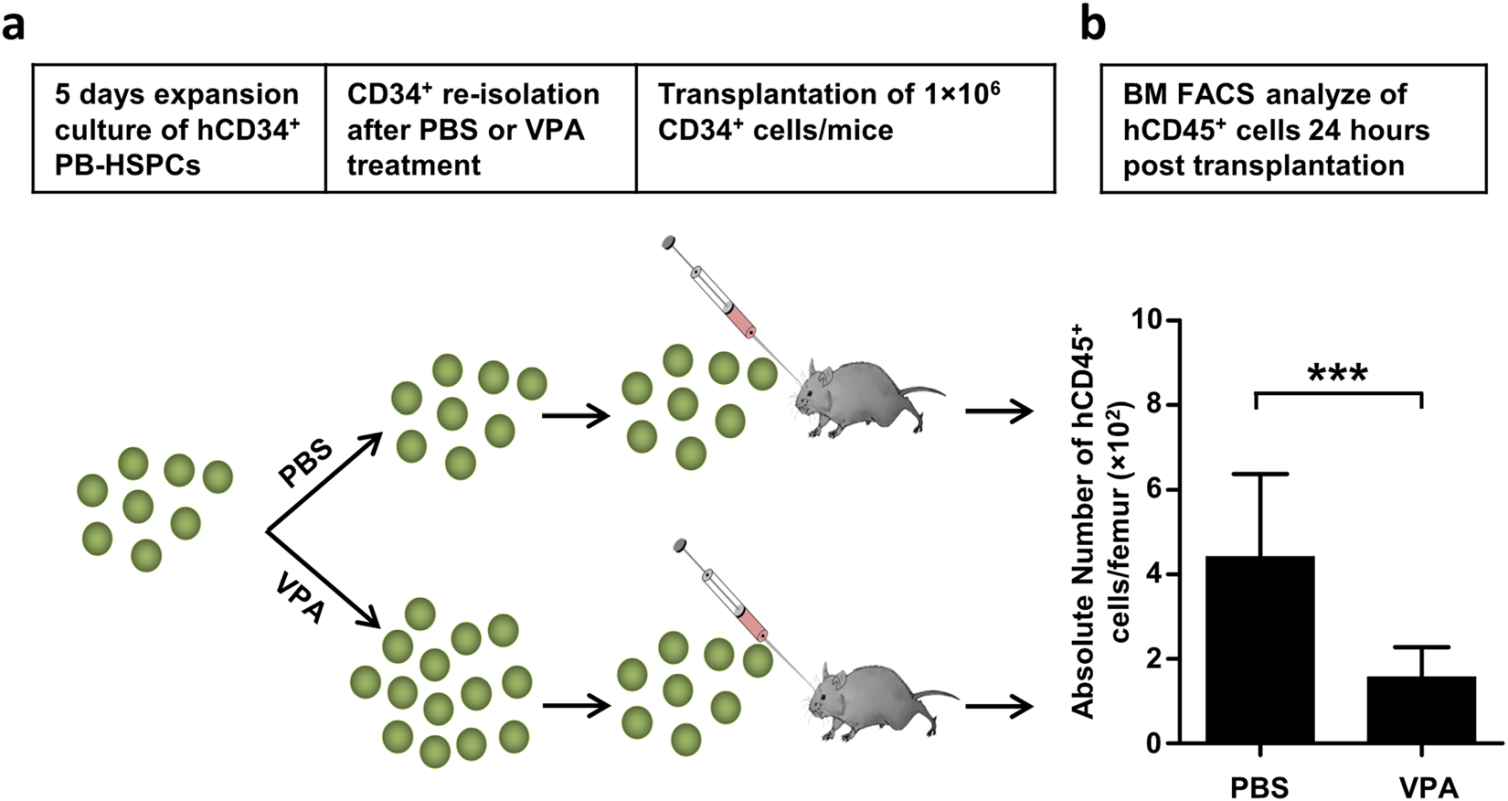
Valproic acid decreases bone marrow homing capacity of *in vitro* expanded CD34^+^ cells. (**a**) Schematic representation of the homing assay performed in NSG mice. CD34^+^ cells were re-isolated after 5 days *ex vivo* treatment with VPA or PBS. 1 × 10^6^ CD34^+^ cells were transplanted by intravenous injection into the retro-orbital venous plexus of sub-lethally irradiated (100 cGy) NSG mice. Homing was quantified by flow cytometric analysis of human leucocytes (human CD45^+^) in the bone marrow of recipient mice 24 hours after injection. (**b**) The absolute number of human leukocytes homing to the femur of recipient mice was significantly reduced by *ex vivo* treatment of CD34^+^ with VPA compared to control cells (n = 8–14). Data are shown as mean ± SD, ***p < 0.001.

As VPA-expanded CD34^+^ cells exhibited reduced migration towards SDF-1 *in vitro*, we tested their bone marrow homing capacity in NSG mice. Equal numbers of re-isolated CD34^+^ cells (1 × 10^6^ cells/mouse), treated with either VPA or PBS, were intravenously injected into sub-lethally irradiated NSG mice (Fig. 6a), and the bone marrow of recipient mice analyzed for the presence of human CD45^+^ cells 24 hours after injection. Mice injected with VPA-treated human CD34^+^ cells had significantly lower number of human leucocytes in the bone marrow compared to those that received control cells (1.6 ± 0.2 × 10^2^/femur vs. 4.4 ± 0.7 × 10^2^, p = 0.0001; Fig. 6b), implying reduced *in vivo* bone marrow homing capacity of these cells.

### VPA-expanded hHSPCs retain their long-term repopulating potential in immunocompromised mice

**Figure 7:**
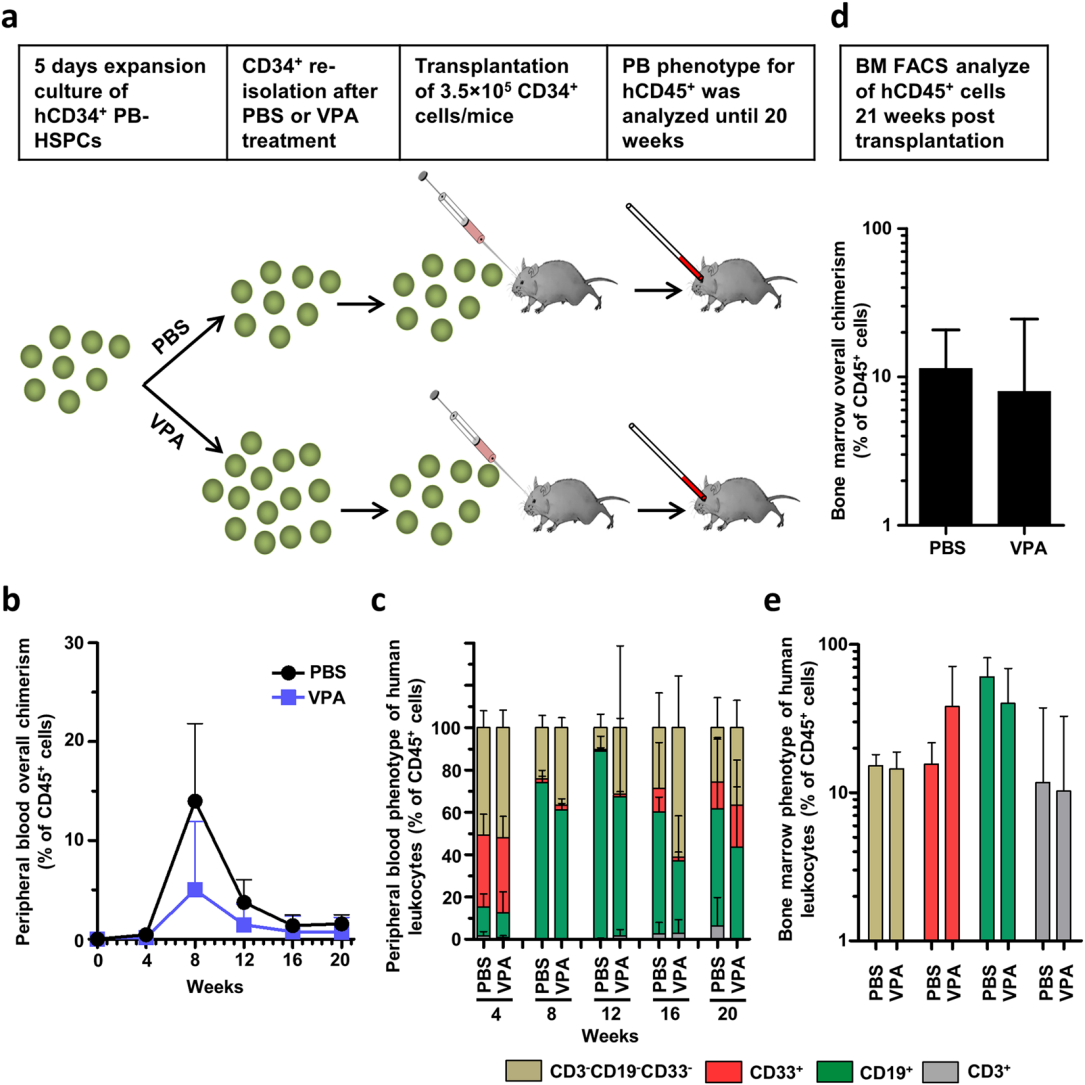
Valproic acid modifies short-term engraftment but does not influence long-term engraftment and differentiation capacity of VPA expanded CD34^+^ cells. (**a**) Schematic representation of the engraftment assay performed in NSG mice. Sub-lethally irradiated (100 cGy) mice were intravenously transplanted with 3.5 × 10^5^ CD34^+^ VPA-treated or control cells. Engraftment of human CD34^+^ cells was monitored by analyzing chimerism and phenotype of circulating human leucocytes (human CD45^+^) in the peripheral blood of recipient mice every four weeks by flow cytometry. Long-term *in vivo* marrow repopulation capacity was determined at 20 weeks after transplantation by quantification and phenotyping of human leucocytes in the femur of NSG mice (n = 5 per group). (**b**) Overall peripheral blood chimerism increased by week 12 and subsequently declined. Mice transplanted with PBS-treated CD34^+^ cells showed elevated overall chimerism compared to mice that received VPA-treated CD34^+^ cells. Differences were most pronounced at week 8 but did not reach statistical significance (p = 0.913). Data represent mean + SD. (**c**) Lineage commitment of circulating human leucocytes was examined by analyzing CD3, CD19, and CD33 cell surface expression with no significant differences in the proportion of T-cells, B-cells or myeloid cells. (**d**) Absolute numbers of human leucocytes (human CD45^+^) per femur at week 20 after transplantation did not differ between groups (p = 0.61). (**e**) Lineage diversification of long-term marrow repopulating human leucocytes was similar in mice injected with VPA-treated or PBS-treated human CD34^+^ cells at 20 weeks after transplantation.

Next, we investigated the repopulating potential of *in vitro* VPA-expanded human CD34^+^ cells by transplanting equal numbers of VPA-expanded or PBS-treated re-isolated CD34^+^ cells into sub-lethally irradiated immunocompromised mice. Peripheral blood chimerism and the phenotype of circulating human leucocytes were monitored periodically and human leucocyte repopulation of the bone marrow was assessed at 20 weeks after transplantation (Fig. 7a). While there were no differences in peripheral blood chimerism at the 4-week time point or the 12-week and later time points, at 8-weeks mice receiving PBS-treated CD34^+^ cells tended to have higher overall chimerism compared to those given VPA-treated CD34^+^ cells (14 ± 3.5% vs. 5 ± 3.1%, p = 0.0913, unpaired t-test) (Fig. 7b). Thus, despite impaired short-term engraftment, as measured by peripheral blood chimerism, the phenotype of the circulating human leucocytes was indistinguishable between the two groups, suggesting that *in vitro* VPA treatment did not affect the differentiation potential of these cells for the main hematopoietic lineages, namely, CD3^+^ T cells, CD19^+^ B cells, and CD33^+^ myeloid cells (Fig. 7c). Similar to the results of peripheral blood chimerism, long-term bone marrow engraftment in recipient mice showed no significant differences in overall BM chimerism (PBS: 11.3 ± 4.2%, VPA: 8 ± 7.4%; Fig. 7d). Further, the numbers of human leucocyte-derived T lymphocytes (CD3^+^), B lymphocytes (CD19^+^), and myeloid cells (CD33^+^) were indistinguishable between the two groups (Fig. 7e). These results confirm *in vitro* data (Fig. S3) that VPA treatment does not negatively affect the long-term engraftment capacity and differentiation potential of human CD34^+^ cells. Importantly, as VPA treatment increases the overall number of CD34^+^ cells by 2–3 fold compared to controls (Fig. 3b), *ex vivo* VPA treatment results in a net expansion of mouse-repopulating HSCs.

### VPA-induced changes in the gene-expression profile of CD34^+^ HSPCs

**Figure 8:**
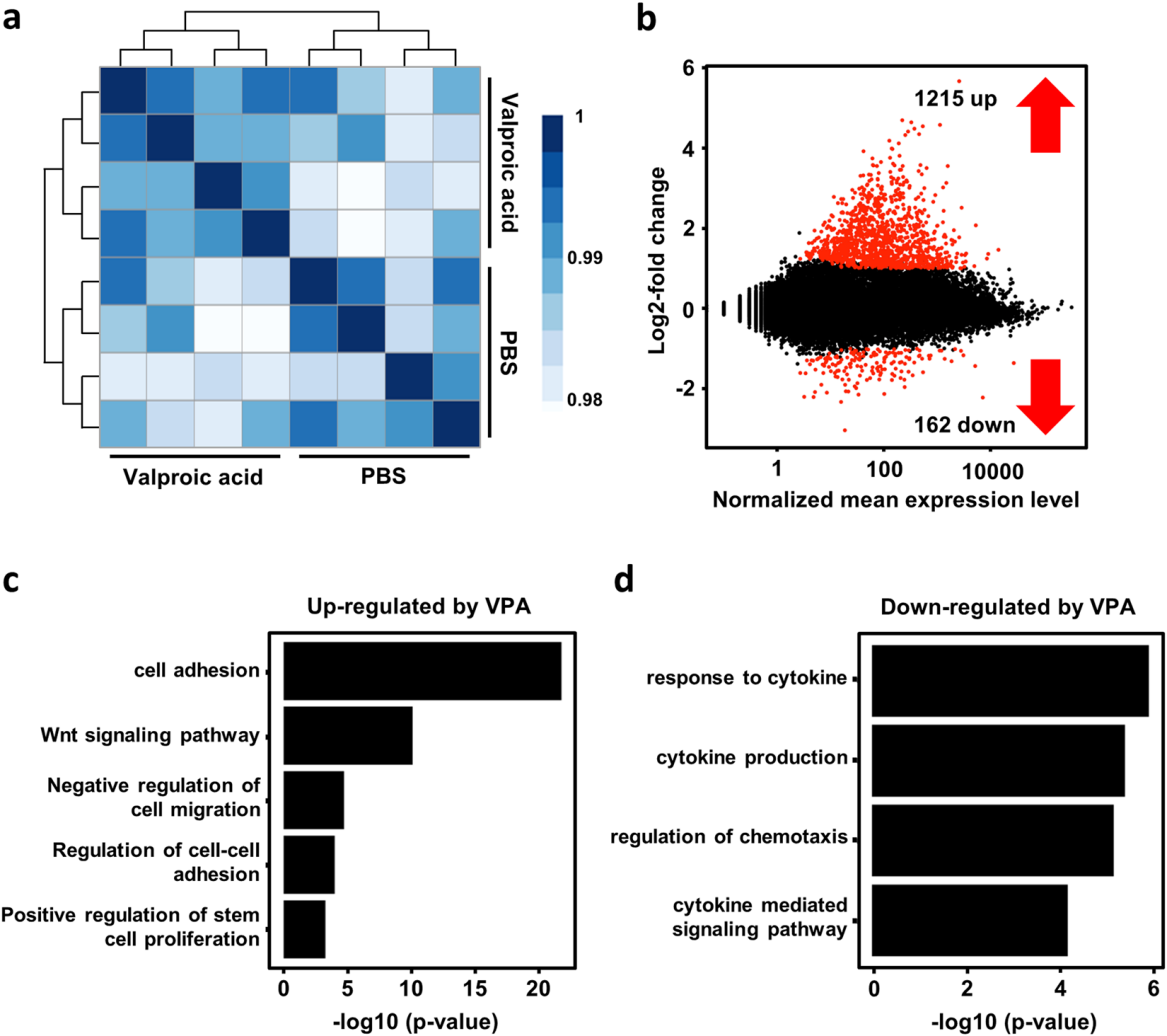
Gene expression profiling revealed induction of cell adhesion pathways and reduced expression of genes involved in chemotaxis. (**a**) Heat map of a sample-to-sample Pearson correlation and dendrogram showing sample-to-sample correlation. All biological replicates cluster well with each other, and all samples from different populations are clearly separated from each other (analysis by R). (**b**) The red dots represent differentially expressed genes (DEG) by VPA treatment (1% false discovery rate). (**c**,**d**) GO pathway analysis for differentially expressed genes. Plots show biological processes that are associated with genes that are up- or down-regulated in VPA treated CD34^+^ cells.

To test how VPA affects gene expression in CD34^+^ cells, we performed mRNA sequencing. Freshly isolated CD34^+^ cells were treated with either VPA or PBS for 5 days and RNA-seq performed on total RNA isolated from both sets of CD34^+^ cells. The clustering of individual biological replicates revealed high homology between samples (Fig. 8a). A comparison of gene expression profiles between the two sets revealed that VPA treatment upregulated 1215 genes and downregulated 162 genes (Fig. 8b). GO (gene ontology) pathway analysis revealed an increase in the expression of genes predominantly involved in cell adhesion due to VPA treatment. This observation is in agreement with data from attachment assays, including AFM-SCFS, which showed greater adhesiveness of VPA-expanded cells toward MSCs. In addition, VPA-treated CD34^+^ cells also significantly downregulated genes that participate in chemotaxis. This result is in line with the observed impairment of chemotactic behavior *in vitro*, especially the SDF-1/CXCR4 axis. VPA-expanded cells also increased the expression of genes involved in Notch and wnt signaling (Fig. S6). Pluripotency genes such as *OCT4*, *SOX2*, and *NANOG* were not upregulated (Fig. S5), and the changes in the expression of *CXCR4*, *MCAM*, *OCT4*, *SOX2*, and *NANOG* were further verified by quantitative real-time PCR.

## Discussion

We have identified HDAC inhibitors, particularly VPA, resminostat, and entinostat, as potent *in vitro* multipliers of hHSPCs. Initially, a transgenic zebrafish model was used to screen for molecules capable of expanding the HSPC pool *in vivo*, and although several HDACIs were included in the screen, only VPA, resminostat, and entinostat increased the number of c-myb^+^ HSPCs, suggesting that HSPC expansion is specific to these three drugs rather than a general group effect of HDACIs. Importantly, the results from the zebrafish screen were reproduced in human HSPCs as *in vitro* treatment of CD34^+^ cells isolated from G-CSF mobilized peripheral blood with these HDACIs led to a significantly greater expansion of both phenotypic and functional HSPCs. Even though *in vitro* VPA-induced expansion of G-CSF mobilized CD34^+^ cells was associated with functional changes that affected homing and short-term engraftment, their *in vivo* long-term engraftment (and thereby self-renewal) and differentiation capacity remained unaltered.

The use of G-CSF mobilized HSPCs has many advantages over allogeneic bone marrow transplantation such as earlier hematopoietic recovery in the recipient, higher anti-leukemic activity, and a less invasive procedure for the donor. Engraftment is also much faster compared to cord blood stem cells and the greater numbers of HSPCs obtained by G-CSF ad apheresis predestine mobilized HSPCs as starting material for future genetic-engineering approaches. Therefore, identification and validation of small molecules that significantly expand mobilized HSPC numbers *ex vivo* represent a clinically attractive approach, and our results imply that *ex vivo* treatment of mobilized HSPCs with VPA can increase the absolute number of CD34^+^ cells with preserved *in vivo* repopulating potential.

While our results have further contributed to the body of evidence that the zebrafish model represents a valuable tool to study vertebrate hematopoiesis^12,28^, more importantly, its use as an *in vivo* screening tool is valuable as large drug libraries can be efficiently screened and any systemic effects made readily apparent.

The fate of any cell, including HSPCs, is substantially influenced by epigenetics^29,30^. Among several functionally relevant epigenetic modifications, DNA methylation and histone acetylation play important roles in health and disease as they regulate gene expression, and drugs that target or modify methylation and acetylation patterns are associated with a range of conditions, including cancer progression^31,32^. Importantly, HDACIs and hypomethylating agents (HMAs) have been shown to alter the epigenetic status of HSPCs^33,34^, and epigenetic modifiers such as 5-Aza 2′-deoxycytidine (an HMA) and trichostatin-A (an HDACI) are capable of expanding CD34^+^CD90^+^ BM cells with preserved long-term repopulating potential^35^. A recent report also showed that VPA can epigenetically reprogram cord blood (CB)-derived CD34^+^ cells *in vitro* that leads to extensive expansion of functional CB HSCs; these cells have improved repopulating capacity in NSG mice compared to control treated cells. Furthermore, they detected that higher number of VPA treated cells residing within G0/G1 and G2/M phase than the cells that are exposed to control^10^. However, they also found that *in vitro* VPA treatment led to greater CXCR4 expression that was associated with enhanced migration toward a SDF-1 gradient *in vitro* and increased homing to the bone marrow of NSG mice *in vivo*. Contrarily, our cell cycle analyses revealed no difference in the proportion of CD34 + cells in G0/G1 vs S/M-phase after VPA treatment compared to control (Fig. S2) and we show that CXCR4 downregulation upon VPA treatment, identified by flow cytometry and confirmed by direct RNA-Seq, and complementary simultaneous upregulation of SDF-1, indicating the presence of an autocrine feedback-loop^36^. These disparities in CXCR4 expression between the two studies can be explained in part by differences in HSPC source and experimental conditions such as cytokine concentration/combination and culture medium. Further, G-CSF mobilized CD34^+^ cells exhibit robust CXCR4 expression levels probably because their metalloproteinase-induced degradation declines during differentiation^37^. Dynamic changes in CXCR4 expression should also be considered as a previous study reported that CXCR4 expression is heterogeneous in the human HSPC compartment and that its stem cell repopulating potential does not necessarily correlate with CXCR4 expression^38^.

The success of HSCT by intravenous infusion of the graft also relies on the homing of HSCs to recipient bone marrow. This process is regulated by multiple molecular events such as cell-cell interaction, migration and adhesion. We observed reduced homing of the VPA-primed CD34^+^ cells that was associated with either unaffected or lower overall peripheral blood chimerism in recipient mice at 8 weeks after transplantation. Conversely, at this time point; Chaurasia *et al*. (2004) found a significant increase in bone marrow overall chimerism in NSG mice transplanted with VPA-treated CB CD34^+^ cells; however, these results cannot be directly compared, as peripheral blood chimerism does not necessarily reflect levels of bone marrow chimerism.

Apart from reduced migration, we observed an increase in the adhesiveness of VPA-treated CD34^+^ cells toward MSCs in co-culture experiments and quantified this force using AFM–SCFS. Cell adhesion is typically mediated by multiprotein complexes that connect individual cells to the extracellular matrix (ECM) and to other cells. Several specialized adhesion molecules such as ICAM-1, NCAM, VCAM-1, and N-cadherin are expressed on subsets of HSCs and are known to be crucial for HSC function and niche interaction^39–42^. The gene expression data reported here show that several molecules known to be involved in cell adhesion, including NCAM, are upregulated in VPA-treated CD34^+^ cells. Unexpectedly, the expression of melanoma cell adhesion molecule (MCAM, CD146) was upregulated in VPA-treated CD34^+^ cells and its surface expression was verified by flow cytometry. This result is surprising as while it is known that MCAM is commonly expressed within the vascular wall, including in the vascular endothelial cells, the vascular smooth muscle cells, and the MSCs^43^, its expression has not been described in HSCs. Further, MCAM expression on MSCs is known to influence the fate of HSCs^44^. MCAM expression has also been demonstrated on subsets of activated T- and B-lymphocytes, mainly in the context of inflammatory conditions^45^. We hypothesize that this induction of MCAM expression is involved in the increased adhesiveness of HSPCs on MSCs and is supported by AFM measurements.

In contrast to the observed reduction in bone marrow homing and short-term engraftment, long-term engraftment and differentiation capacity of VPA-treated CD34^+^ cells was not different from controls. Also, the relative and absolute numbers of human phenotypic HSPCs in the bone marrow of NSG mice at 20 weeks after transplantation were indistinguishable between VPA and controls, suggesting that the *in vitro* VPA-expanded CD34^+^ cells retained their stem cell properties. These results show that VPA significantly influences the functional activity of HSPCs but the effect on stem cell frequency still needs to be determined. A previous report showed that VPA-induced expansion of CB CD34^+^ cells *in vitro* was associated with a transient increase in the expression of genes associated with chromatin remodeling and pluripotency in induced pluripotent stem cells (iPS) and embryonic stem cells (ES), such as *SOX2*, *OCT4*, and *NANOG* ^10^. Interestingly, we found that VPA did not induce the expression of these pluripotency genes in G-CSF mobilized adult CD34^+^ cells (Fig. S5) but rather upregulated *Jag2*, *Notch3*, *Hes1*, and *DLL1* (Fig. S6a). Such recurrent variations in results between these two studies point to the differential effects of VPA on the ontogenetically more immature CB CD34^+^ HSCs compared to the mature G-CSF mobilized adult CD34^+^ cells. Importantly, these genes (*Jag2*, *Notch3*, *Hes1*, and *DLL1)* and their related pathways are all known to be involved in the preservation of stem cell properties during the *ex vivo* expansion of HSCs^46^. Furthermore, *DLL1* induces erythroid differentiation and inhibits myeloid skewing^47,48^.

The role of wnt signaling in the regulation of hematopoiesis has been extensively studied^49^, and both the canonical and non-canonical pathways of wnt signaling are crucial for HSC maintenance and activation^50^. Gene expression data presented here (Fig. S6b) show upregulation of several genes that are involved in both canonical and non-canonical wnt signaling, such as Frizilled (Fzd) 8 and Flemingo (Fmi), members of the non-canonical wnt signaling pathway that are crucial for long-term quiescent HSC maintenance^51^.

To conclude, we show that certain HDACIs enhance the *in vitro* expansion of CD34^+^ HSPCs derived from G-CSF mobilized peripheral blood and that, functionally, these cells are capable of effective long-term engraftment and multi-lineage differentiation, even though VPA treatment led to reduced homing and short-term engraftment that are probably related to lower migratory capacity and enhanced adhesiveness. Importantly, as our observations are in contrast to previous work with cord blood derived CD34^+^ cells, we conclude that VPA-mediated effects on HSPCs are distinct and depend on the graft source used. Lastly, impaired short-term engraftment that might lead to prolonged cytopenia after transplantation of *in vitro* VPA expanded G-CSF mobilized peripheral blood stem cells has to be considered when translating HDACI-based expansion protocols into the clinical setting.

## Materials and Methods

Detailed materials and methods are described in the supporting information materials and methods. All experiments were performed in accordance with the local and national regulations and guidelines and were approved by the local institutional review board (Ethical committee of Technischen Universität Dresden, Fiedlerstraße 33, 01307 Dresden). The xenotransplant experiments were performed according to the national guidelines after approval by the local animal protection committee.

### Small molecule screen and validation

All animal experiments were carried out in accordance with live animal handling and research regulations under protocols approved by the animal ethics committees of the Technische Universität Dresden and the Landesdirektion Sachsen (approval number no: AZ 24D-9168.11-1/2008-4). Fish were maintained at 28 °C^52^, and synchronized embryos from homozygous zebrafish *c-myb*:EGFP^12^ transgenic line were collected in E3. The semi-automated *in vivo* chemical screen was performed as described previously^18^. Several small focused libraries were screened, including an epigenetic modulator library (Selleckchem) containing several HDACIs. Identified hits from the chemical screen were validated by whole-mount *in situ* hybridization for *runx1* expression as described elsewhere^53^. Compounds were also tested at a concentration of 40 µM and imaged as reported previously^54^.

### Adult zebrafish kidney marrow transplantation

Adult kidney marrow cells from multiple *Tg(ubi:GFP)* fish were harvested, WKM cells dissociated, pooled, and filtered^55^. Unfractionated WKM cells were incubated *in vitro* at 28 °C in ZKS (zebrafish kidney stromal) medium^56^ and treated with either VPA (50 µM) or PBS control for 48 hours. One day before transplantation, three-month-old Casper recipient fish were sub-lethally irradiated at 15 cGy. The *ex vivo* chemical treatment of WKM cells and their transplantation were performed as previously reported^57^ (Fig. S1). Fresh WKM cells from *Tg(ubi:GFP)* animals were used as a positive control to measure engraftment efficiency. Donor chimerism was assessed on a BD LSR II flow cytometer (BD Biosciences) and its analysis limited to myelomonocytes, as described previously^58^.

### *Ex vivo* culture and co-culture assay

G-CSF (granulocyte colony-stimulating factor) mobilized peripheral blood for CD34^+^ HSPC isolation and bone marrow for MSC isolation was obtained from healthy donors after informed consent (ethical approval no. EK221102004, EK47022007) and human cells were isolated according to the Ethical committee of Technischen Universität Dresden (https://www.tu-dresden.de/tu-dresden/organisation/gremien-und-beauftragte/kommissionen/ethikkommission). Purified CD34^+^ cells (1 × 10^4^ cells/well) were cultured in CellGro serum free medium (CellGenix) supplemented with 10 ng/ml stem cell factor (SCF), 10 ng/ml Fms-related tyrosine kinase 3 ligand (FLT3 ligand), 10 ng/ml interleukin 3 (IL-3), and incubated at 37 °C in 5% CO_2._ After 24 h, the cells were exposed to PBS, VPA (1 mM), DMSO, resminostat (1.5 µM), or entinostat (1.5 µM) for 5 days. After treatment, expanded cells were stained for CD34 and CD90 expression. For further experiments, CD34^+^ cells were re-isolated from *ex vivo*-expanded cells and their purity analyzed as stated above. For co-culture experiments, freshly isolated CD34^+^ cells were suspended in CellGro serum free medium supplemented with cytokines and plated onto a confluent MSC layer. Co-culture of CD34^+^ cells with human MSCs was performed as previously described^11^.

### Atomic force microscopy-based single-cell force spectroscopy (AFM-SCFS)

A NanoWizard II AFM equipped with a CellHesion module (JPK Instruments), mounted on an inverted light microscope (Axiovert 200, Carl Zeiss), was used to perform AFM-SCFS. All measurements were performed in PBS containing Ca^2+^/Mg^2+^ at 37 °C using a temperature-controlled sample chamber (PetriDish Heater, JPK Instruments). Tipless cantilevers with a nominal spring constant of 0.08 N/m (PNP-TR-TL-Au, Nanoworld) were coated with 1 mg/ml wheat germ agglutinin (Vector Laboratories) as described previously^59^. Cantilevers were calibrated before every experiment using built-in functions of the AFM software (JPK Instruments) based on the equipartition theorem^60^. For SCFS experiments, MSCs were grown to confluence in 30-mm Petri dishes (TPP-Sigma-Aldrich). The Petri dish was placed in the PetriDish Heater and subsequently, 1 × 10^3^ CD34^+^ HSPCs, either PBS- or VPA-treated for 5 days, were seeded into the Petri dish. A single HSPC was attached to the AFM cantilever as described^59^. Force-distance (F-D) curves were acquired by approaching the cantilever-bound HSPC at a constant speed of 5μm/sec onto a single MSC in the Petri dish until a contact force of 2 nN was reached. Before retraction of the cantilever-bound cell at constant speed (5 μm/sec), cells were maintained in contact for 60 sec in constant force mode. Approximately 10 F-D curves per cell and 10 cells/condition were measured, yielding 100 data points per condition. Data processing software provided by the AFM manufacturer was used to extract maximum detachment force (F_detach_) from retract F-D curves.

### Homing Assay

All animal experiments were performed according institutional guidelines and the German animal protection law (Landesdirektion Sachsen, 24-9168.11-1/2013-54). Re-isolated CD34^+^ cells (1 × 10^6^ cells/mouse), both VPA-treated and controls, were transplanted by retro-orbital injection into NSG mice. The recipient mice were irradiated (100 cGy) 24 h prior to transplantation. Bone marrow of recipient mice (one femur/mouse) was analyzed 24 h after transplantation by flow cytometry for the presence of human CD45^+^. Eight NSG mice received PBS expanded cells (control) while 14 mice received VPA-expanded cells. Eleven mice did not receive any cells and their bone marrow was similarly analyzed to determine thresholds for the analysis of human cells.

### Long-term engraftment assay

CD34^+^ cells were re-isolated after 5 days *ex vivo* treatment with VPA or PBS (control) and 3.5 × 10^5^ cells/mouse were transplanted by intravenous injection in the retro-orbital venous plexus of the recipient mice. NSG mice were sub-lethally irradiated with 100 cGy 24 h prior to transplantation. To assess engraftment, peripheral blood from recipient mice was obtained every 4 weeks by retro-orbital bleeding and collected in heparinized hematocrit capillaries (Brand GMBH). Peripheral blood was analyzed by flow cytometry using anti-mouse CD45, anti-human CD45, CD3, CD19 and CD33 antibodies. Mice were sacrificed at 20 weeks after transplantation and the bone marrow of each mouse (2 femurs and 1 tibia) was analyzed for the presence of human cells using anti-mouse CD45, anti-human CD45, CD3, CD19, and CD33 antibodies.

### Statistics

Data were analyzed using student’s t-test by GraphPad Prism 6 (GraphPad Software, Inc.). All data are from at least three independent experiments, unless otherwise specified. Results are expressed as the mean ± SD or the mean ± SEM of varying numbers in individual experiments. Statistical significance was defined as *P < 0.05; **P < 0.01; ***P < 0.001.

### Data availability

The authors declare that the data supporting the findings of this study are available within the paper and its supplementary information files or can be obtained from the authors upon reasonable request. The gene expression data reported in this paper have been deposited in the Gene Expression Omnibus (GEO) database, www.ncbi.nlm.nih.gov/geo (Reference super-series GSE90552).

## Electronic supplementary material

Supplementary Information
