# Supplementary material for: Zebrafish In-Vivo Screening for Compounds Amplifying Hematopoietic Stem and Progenitor Cells: - Preclinical Validation in Human CD34+ Stem and Progenitor Cells
Source: Sci Rep. 2017 Sep 21;7:12084. doi: 10.1038/s41598-017-12360-0 (PMC5608703; doi:10.1038/s41598-017-12360-0)
Supplement: Supplementary file 1 — Supplementary Information [file 41598_2017_12360_MOESM1_ESM.pdf]

Zebrafish In-Vivo Screening for Compounds Amplifying Hematopoietic Stem and Progenitor Cells: - Preclinical Validation in Human CD34+ Stem and Progenitor Cells

Authors:

Guruchandar Arulmozhivarman[1;$], Martin Kräter[1;$], Manja Wobus[1], Jens Friedrichs[2], Elham Pishali Bejestani[3;4], Katrin Müller[1], Katrin Lambert[1], Dimitra Alexopoulou[5], Andreas Dahl[5], Martin Stöter[6], Marc Bickle[6], Nona Shayegi[7], Jochen Hampe[1], Friedrich Stölzel[1], Michael Brand[8;*], Malte von Bonin[1;3;4; $], Martin Bornhäuser[1;8;*; $]

Supplementary information

Supplemental Methods

Isolation of HSPCs and MSCs

$CD34^+$ hematopoietic stem and progenitor cells (HSPC) were purified from G-CSF mobilized peripheral blood obtained from healthy donors who were treated with 7.5 µg/kg granulocyte colony-stimulating factor for 5 consecutive days. Informed consent was obtained in accordance with a research protocol approved by the local institutional review board. $CD34^+$ HSPCs were enriched via magnetic affinity cell sorting (MACS) using CD34 antibody-conjugated magnetic beads according to manufacturer's instructions (Miltenyi Biotec). The purity of the isolated $CD34^+$ cells was evaluated by flow cytometry and ranged from 95 - 99%. MSCs were obtained from healthy donors after informed consent (ethical approval no.: EK263122004). Density gradient centrifugation (mononuclear fraction) and plastic adherence capacity were used to isolate MSCs, as described elsewhere [1], and their purity was confirmed by flow cytometry ($CD45^-$, $CD34^-$, $CD73^+$, $CD90^+$, $CD105^+$, and $CD166^+$). MSCs were cultured in DMEM GlutaMax (Invitrogen) supplemented with 10% fetal bovine serum (FCS; Biochrom).

Flow cytometry

Flow cytometry was performed on a BD FACS Calibur (*in vitro* experiments), BD LSR II (*in vivo* experiments) (BD Biosciences) or MACSQuant (*in vivo* experiments) (Miltenyi Biotec), as required. The following antibodies were used in various combinations: antihuman CD34-FITC (clone: AC136, Miltenyi Biotec), antihuman CD90-APC (clone: eBio5e10, eBioscience), antihuman CD146-APC (clone: 541-10B2, Miltenyi Biotec), antihuman CD184 (clone: 12G5, BD Biosciences), antihuman CD73-PE (clone: AD2, Miltenyi Biotec), antihuman CD105-APC (clone: 43A4E1, Miltenyi Biotec), antihuman CD166-PE (clone: 3A6, BD Biosciences), antimouse CD45-PE (clone: 30F-11, eBioscience), antihuman CD45-VE500 (clone: HI30, BD Biosciences), antihuman CD3-APC-eFluor780 (clone: UCHT1, eBioscience), antihuman CD19-PE-Cy7 (clone: SJ25C1, BD Biosciences), and antihuman CD33-FITC (clone: WM53, eBioscience). DAPI (4',6-diamidino-2-phenylindole, final concentration 20 ng/ml) and PI (propidium iodide, final concentration 1 ng/ml) were used as viability dyes for all *in vivo* experiments. Doublet discrimination was routinely carried out. Data analysis was performed using FlowJo (FlowJo, LLC).

Cell cycle analysis

To separate G0, G1 and S/G2/M phase cells HSPCs were cultured for 5 days under VPA or control conditions, washed once in PBS and AB stained for CD34 (130-090-954 miltenyi). PBS + 5% FCS were used to wash cells, followed by 2% PFA fixation (15 min) and 0.1% Tween20 permeabilization (10 min).Cells were incubated for 2h at RT with Anti-Ki67 AB (ab16667 abcam). After another washing step cells were incubated 30 min at RT using a secondary FITC

labeled AB (F-0382 sigma) and DNA was counterstained using PI. Cells were analyzed using flow cytometer LSRII (BD).

Quantitative real-time PCR

Total RNA was isolated from re-isolated $CD34^+$ cells using the TRIzol reagent (Life Technologies). Single strand cDNA was synthesized using oligo-dT primers provided with the RevertAid First-Strand cDNA synthesis kit according to manufacturer's instructions (Thermo Scientific). Quantitative real-time PCR was performed using a SYBR Green Master mix (Thermo Scientific) on an ABI7500 Fast Real-Time PCR system (Life Technologies). GAPDH was used as the reference gene, and gene specific primer sets are listed in supplementary Table 1. All primers were synthesized by Life Technologies (Thermo Fisher Scientific).

HSPC adhesion assay

MSCs (50,000 cells /well) were seeded onto a 24-well plate in DMEM/10 % FCS and cultured to 80–90 % confluency. HSPCs, both VPA-treated and controls were labeled for 60 min with *Cell Tracker Red CMTPX* (Invitrogen) at 37 °C and 5% CO_2. Before seeding HSPCs (2 x 10^5 cells per well) onto MSCs, MSCs were washed twice with PBS to remove FCS and dead cells. HSPCs and MSCs were incubated for 3 h in CellGro medium containing cytokines for adhesion; all experiments were performed in duplicate. After stringent washing of the cultured cells, cells were fixed with 4% formalin for 20 min at room temperature, washed with PBS, and the adherent red fluorescent HSPCs counted in a fluorescence microscope. For quantification, 10 pictures at 100× magnification were taken in a defined order for each condition and the HSPCs were enumerated using *ImageJ* [2].

Colony-forming cell and cobblestone area-forming cell assay

Colony-forming cell assay was performed in semisolid medium as previously described [3]. Briefly, either fresh or re-isolated $CD34^+$ cells (1×10^3 cells) after 5 days of VPA or PBS (control) treatment were suspended in 3 ml human Stem MACS medium (Miltenyi). Aliquots of 1 ml were plated in 3 petri dishes (35x10 mm; Greiner Bio-One) and cultivated at 37 °C under a humidified 5% CO_2 atmosphere. After 14 days, each well was scored for the number of burst-forming units – erythroid (BFU-E), colony forming units – granulocyte (CFU-G), CFU – granulocyte macrophage (CFU-GM), and CFU – macrophage (CFU-M) according to defined criteria [4].

Cobblestone area-forming cell (CAFC) assay was performed on a human MSC feeder layer. Twenty thousand MSCs per cm^2 were seeded in 24-well plates (Corning) in DMEM containing 10 % FCS 24 h prior to co-culture. For co-culture, the MSC layer was washed once with PBS, and 5 x 10^2 $CD34^+$ HSPCs/ cm^2 were added to the MSC layer in 2 ml LTC-IC medium (Myelocult H5100, Stemcell Technologies) containing 10^{-6} M hydrocortisone (Miltenyi). One-half of the medium was changed weekly. Co-cultures were maintained at 37°C under a humidified 5% CO2 atmosphere in triplicates for 21 days. Cobblestone forming areas/units beneath the MSC layer were defined as small (5-10 cells), medium (11-25 cells) or large (> 26 cells) and microscopically counted.

In the adhesion assay, HSPCs pretreated with either VPA or PBS (control), were seeded on a confluent MSC layer and the number of attached HSPCs was quantified 3 h later.

Transwell migration assay

The migration potential of either VPA-treated or control cells were tested using 5 μm pore size Trans-wells (Corning). Re-isolated $CD34^+$ cells (1×10^5) were washed, re-suspended in 100 μl CellGro medium, loaded in the upper chamber of the Transwell, and 500 μl medium containing recombinant human SDF-1 (100 ng/ml, PeproTech) was placed in the lower well. After incubation for 3 h at 37°C, migrated $CD34^+$ cells were counted by flow cytometry.

Bioinformatic Analysis

RNA libraries were prepared following standard protocols for the NEBNext® Ultra Directional RNA Library Prep Kit (Illumina). Libraries were pooled and sequenced on an illumina® HiSeq 2500, and resulted in ca. 27 – 56 million fragments.

FastQC (http://www.bioinformatics.babraham.ac.uk/) and RNA-SeQC (v1.1.8) were used to perform basic quality control of the sequenced fragments. Alignment of the fragments to human reference genome (release hg38) was done using GSNAP (v2015-12-31), and Ensembl annotation 81 was used to detect fragments spanning splice sites [5]. The uniquely aligned fragments were counted with featureCounts (v1.5.0) and the same Ensembl annotation [6]. Raw counts were normalized based on library size and tested for differential gene expression between the two conditions, PBS and valproic acid, using the DESeq2 R package (v1.10.1) [7]. Accepting a maximum of 1% false discovery rate ($p_{adj} < 0.01$) for the comparison, a fold change of 2 (log2-fold change) was considered as up or down regulation.

References:

1. Oswald, J. *et al.* Mesenchymal stem cells can be differentiated into endothelial cells in vitro. *Stem Cells* **22,** 377–384 (2004).

2. Jellinghaus, S. *et al.* Ephrin-A1/EphA4-mediated adhesion of monocytes to endothelial cells. *Biochim. Biophys. Acta - Mol. Cell Res.* **1833,** 2201–2211 (2013).

3. Alakel, N. *et al.* Direct contact with mesenchymal stromal cells affects migratory behavior and gene expression profile of CD133+ hematopoietic stem cells during ex vivo expansion. *Exp. Hematol.* **37,** 504–513 (2009).

4. Pereira, C., Clarke, E. & Damen, J. Hematopoietic colony-forming cell assays. *Methods Mol. Biol.* **407,** 177–208 (2007).

5. Wu, T. D. & Nacu, S. Fast and SNP-tolerant detection of complex variants and splicing in short reads. *Bioinformatics* **26,** 873–881 (2010).

6. Liao, Y., Smyth, G. K. & Shi, W. FeatureCounts: An efficient general purpose program for assigning sequence reads to genomic features. *Bioinformatics* **30,** 923–930 (2014).

7. Love, M. I., Huber, W. & Anders, S. Moderated estimation of fold change and dispersion for RNA-seq data with DESeq2. *Genome Biol.* **15,** 550 (2014).

Figure Legends

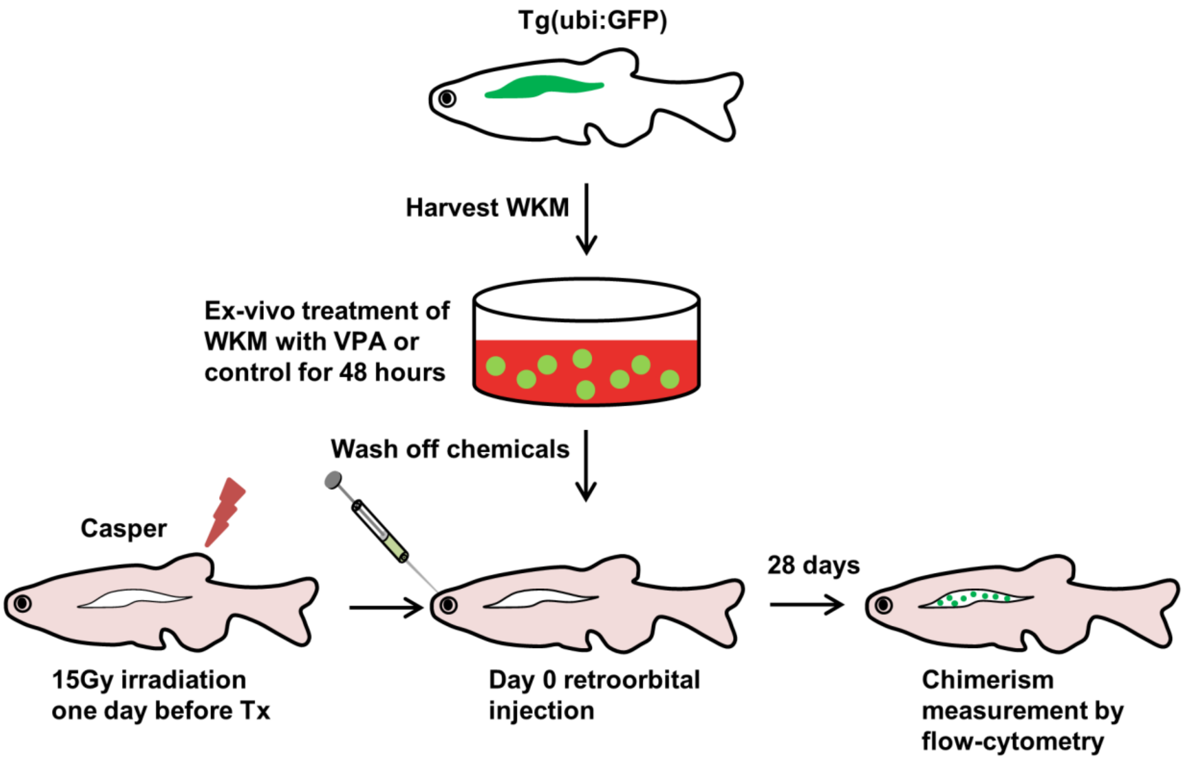

Figure S1

The engraftment potential of zebrafish WKM cells was preserved after VPA treatment.
WKM was dissected from *Tg(ubi:GFP)* donors, dissociated into a single-cell suspension, and treated with VPA for 48 hours. After washing, the cells were resuspended in Tx buffer and retro-orbitally injected into sub-lethally irradiated Casper zebrafish recipients (n = 10 per group). Fresh WKM cells were transplanted into another group of irradiated Casper fish and used as a positive control for quantification. Recipient WKM was dissected and analyzed for donor chimerism at 28 days after transplantation.

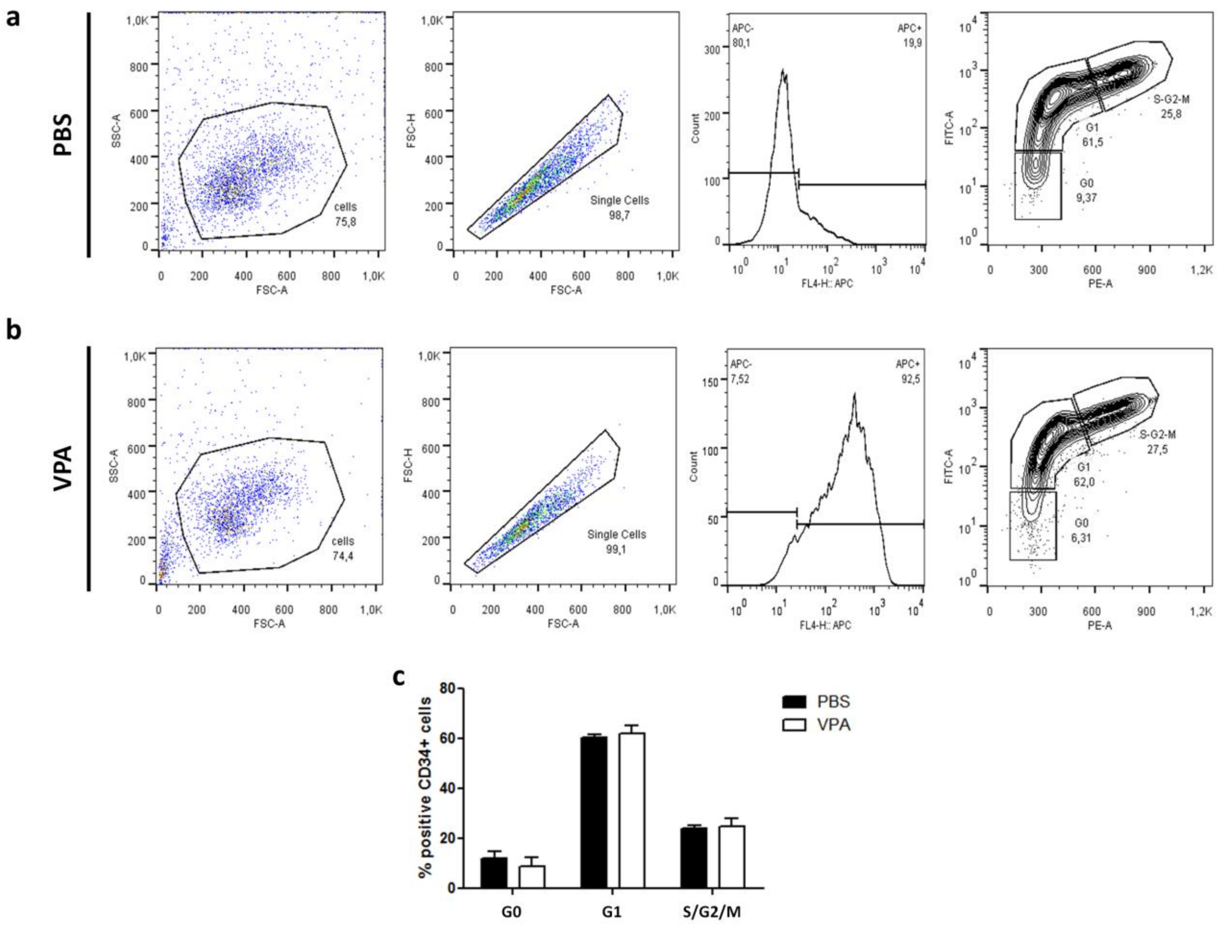

Figure S2

Cell cycle analysis of CD34+ cells treated with PBS vs VPA for 5 days. (A-B) Histograms showing the percentage of $CD34^+$ cells after PBS and VPA treatment. The dot plots showing cells in different phases of the cell cycle between PBS and VPA conditions. (C) Percentage of PBS and VPA treated cells that were in different phases of the cell cycle. No significant differences found between PBS vs VPA conditions (N=3).

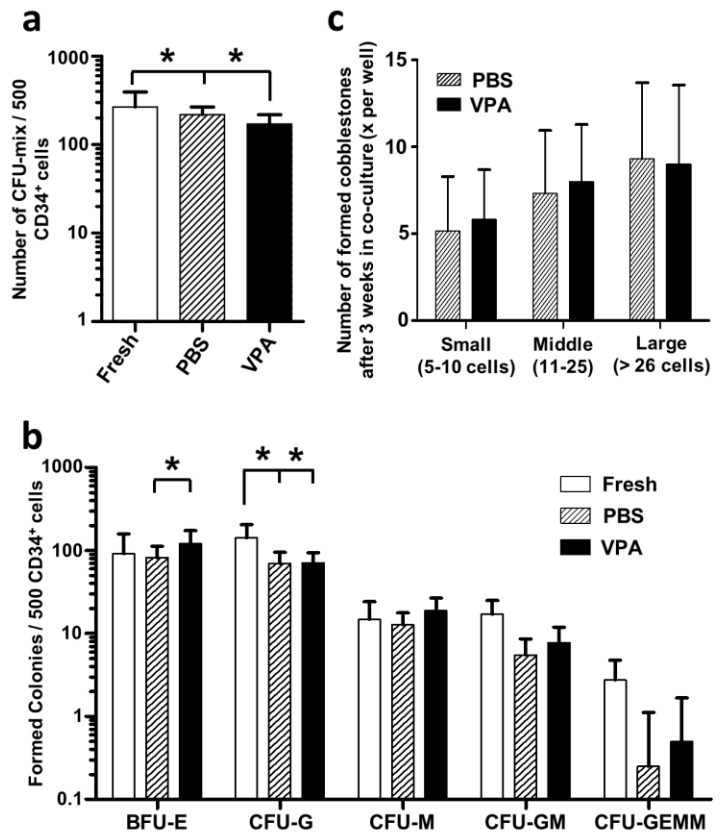

Figure S3

Differentiation potential and clonogenicity of VPA and control treated CD34$^+$ cells *ex vivo*.

To test differentiation potential and stem cell properties of freshly isolated human CD34$^+$ cells and to compare them to 5 day VPA treated or control re-isolated CD34$^+$ cells, colony forming unit assay and cobblestone area forming cell assay (CFAC) were performed. **(A)** The total number of colonies formed by CD34$^+$ cells was significantly lower in the VPA treated group. **(B)** The difference in total number of colonies between the VPA and PBS treated group was mainly caused by an increase in BFU-E capacity in PBS treated CD34$^+$ cells. In contrast, freshly isolated CD34$^+$ cells produced significantly more CFU-G, CFU-GM, and CFU-GEMM colonies compared to those expanded *in vitro*. There were no statistically significant differences in the

total number of CFU-G, CFU-M, CFU-GM, and CFU-GEMM between VPA treated and PBS treated $CD34^+$ cells (n = 3). **(C)** In the CFAC assays using MSCs as feeder layer VPA treated or control $CD34^+$ cells performed equally when followed for up to 3 weeks (n = 4). Data are mean ± SD, $*p < 0.05$.

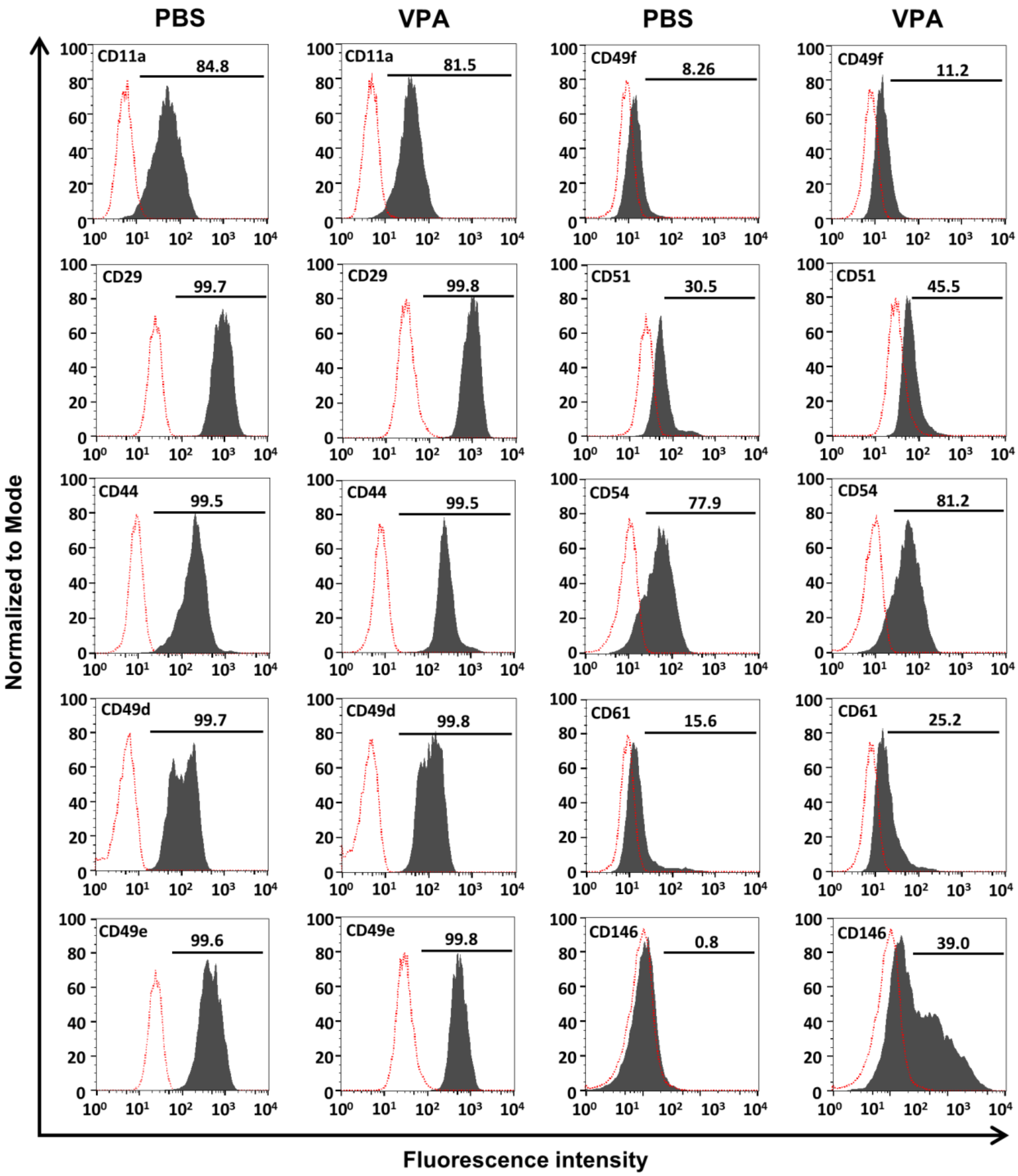

Figure S4

VPA treatment of human CD34$^+$ cells *in vitro* altered surface expression profile of cell adhesion molecules. To determine the mechanism leading to a higher adhesive phenotype in

VPA treated $CD34^+$ cells, the expression of various cell surface adhesion molecules (CD11a, CD29, CD41, CD44, CD49d, CD49e, CD49f, CD54, CD61, and CD146) was determined by flow cytometry after 5 days of *ex vivo* treatment with VPA. There were no statistically significant differences in the proportion of $CD34^+$ cells expressing CD11a, CD29, CD44, CD49d, CD49e, CD49f, CD54, or CD61. VPA-treated cells showed an up-regulation of CD146 (MCAM) expression.

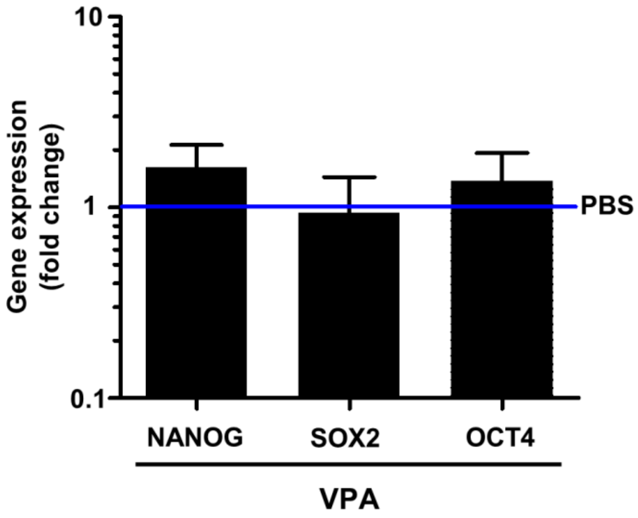

Figure S5

Valproic acid treatment of human CD34$^+$ cells *in vitro* does not alter the expression of pluripotency genes compared to control cells. The expression of key pluripotency genes (*oct4*, *sox2*, and *nanog*) in *ex vivo* expanded CD34$^+$ cells was determined by quantitative real-time PCR. CD34$^+$ cells were re-isolated after 5 days treatment with VPA or PBS, total RNA isolated, and cDNA prepared. Relative change in gene expression of VPA-treated cells was compared to control cells (n = 3). Data are mean ± SD.

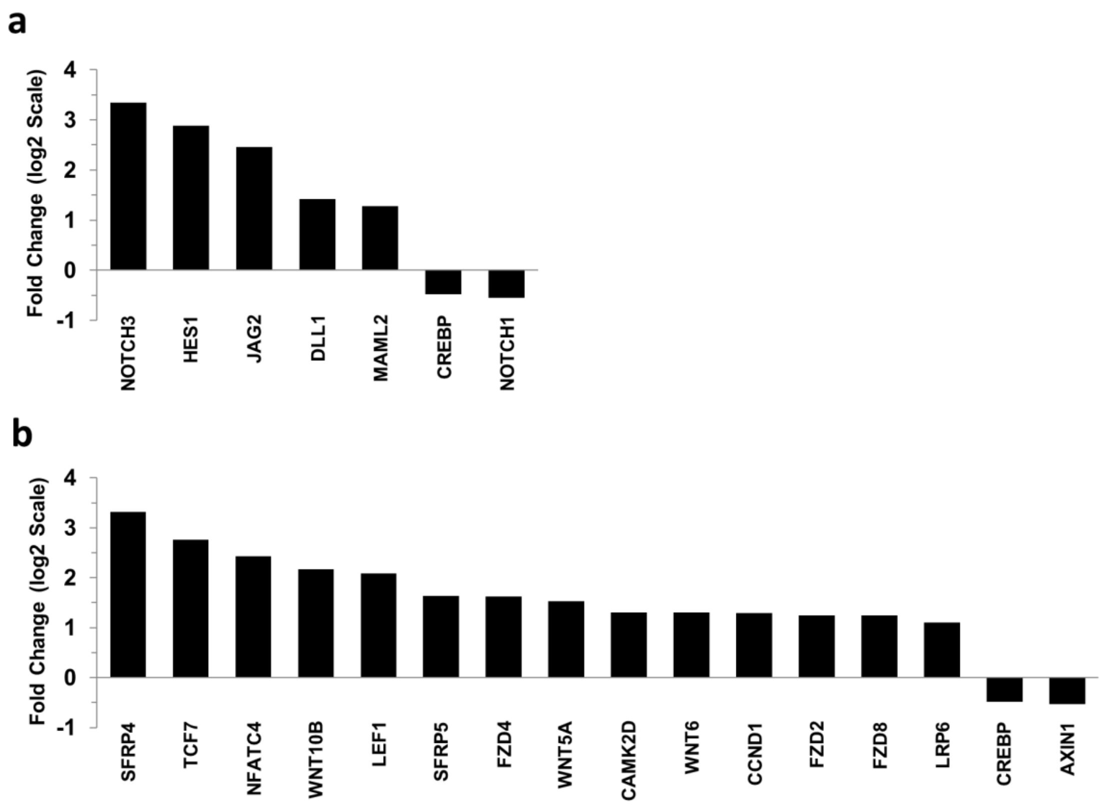

Figure S6

RNA-sequencing analysis to measure changes in gene expression level in CD34$^+$ cells after 5 days treatment of with VPA. (A) Bar plot shows Notch-signaling pathway genes that were either upregulated or downregulated by VPA treatment compared to control CD34$^+$ cells (n = 4). **(B)** Bar plot displays Wnt pathway-related genes that are upregulated or downregulated by VPA treatment compared to control CD34$^+$ cells (n = 4).

Supplementary Table S1: List of primer sequence used for qPCR

Gene	Forward Primer Sequence	Revers Primer Sequence
**GAPDH**	GAAGGTGAAGGTCGGAGTC	GAAGATGGTGATGGGATTTC
**CXCR4**	CTCTATGCTTTCCTTGGAGCC	TGGACCCTCTGCTCACAG
**CD146**	CTGCTGAGTGAACCACAGGA	CACCTGGCCTGTCTCTTCTC
**OCT4**	AACCTGGAGTTTGTGCCAGGGTTT	TGAACTTCACCTTCCCTCCAACCA
**SOX2**	AGAAGAGGAGAGAGAAAGAAAGGGAGAGA	GAGAGAGGCAAACTGGAATCAGGATCAAA
**NANOG**	CCTGAAGACGTGTGAAGATGAG	GCTGATTAGGCTCCAACCATAC

Supplementary Table S2: Name of the positive and negative hits

Positive Hits	Negative Hits
Valproic Acid, Sodium Salt	Adenosine Kinase Inhibitor
Entinostat (MS-275, SNDX-275)	(±)-Bay K 8644
Mitochondrial Division Inhibitor, mdivi-1	Lithium Chloride
Mocetinostat (MGCD0103)	
Resminostat hydrochloride	
Hh/Gli Antagonist, GANT61	
5-Aza-2′-Deoxycytidine	

Supplementary Table S3: Number of animals that engrafted per condition and their engraftment level

Fish ID	PBA (%)	VPA (%)
1	0.7	65.2
2	1.3	2.3
3	1.5	63.2
4	0.5	68.5
5	2.7	
